# Published population pharmacokinetic models of mycophenolate sodium: a systematic review and external evaluation in a Chinese sample of renal transplant recipients

**DOI:** 10.3389/fphar.2025.1632568

**Published:** 2025-08-18

**Authors:** Tong Gao, Wen Xu, Xiao Li, Qie Guo, Donghua Liu, Xiaolei Zhang, Ping Leng, Jialin Sun

**Affiliations:** Department of Pharmacy, The Affiliated Hospital of Qingdao University, Qingdao, China

**Keywords:** population pharmacokinetics, mycophenolate sodium, external evaluation, renal transplant recipients, therapeutic drug monitoring

## Abstract

**Background:**

Immunosuppressive therapy remains the primary method for preventing rejection in renal transplant recipients. While multiple population pharmacokinetic (popPK) models of mycophenolate sodium (MPS) have been developed for this population, their predictive performance across different clinical settings remains unverified. This study systematically evaluated published MPS popPK models through external validation to assess their extrapolation potential.

**Methods:**

Published MPS popPK models for renal transplant recipients were identified through systematic searches of PubMed, Embase and Web of Science. These models were externally evaluated using a cohort of renal transplant patients receiving MPS therapy at the Affiliated Hospital of Qingdao University. Model prediction performance was evaluated using three metrics: the goodness-of-fit method based on model prediction, prediction error test method and visual predictive checks method based on model simulation.

**Results:**

A total of 186 drug concentration data of 31 patients in our hospital were collected, and 4 literature were retrieved, among which 1 were one-compartment models and 3 were two-compartment models. In the goodness-of-fit diagnosis and prediction error test based on model prediction, the population prediction data of all models were not good, while the individual prediction data showed that the fitting result of Model 1 was relatively better. The visual prediction test results based on model simulation show that the fitting result of Model 1 was relatively good, while the distribution deviation between the observed data and the simulation data of the remaining models was large, and the fitting effect was not good.

**Conclusion:**

The published models exhibit significant variability and unsatisfactory predictive performance, indicating that therapeutic drug monitoring (TDM) remains an essential requirement for the clinical application of MPS. To advance individualized medication for MPS based on popPK, future research must prioritize the investigation of potential covariates. This will enable identification of key factors influencing MPS model predictability and facilitate the development of a popPK model suitable for patients in our hospital.

## 1 Introduction

Renal transplantation, as the most ideal renal replacement therapy for patients with end-stage chronic renal failure, has gained widespread recognition in the global field of organ transplantation. However, long-term prognosis management continues to pose significant challenges (Hariharan et al., 2021; Wolfe et al., 1999; Zhao et al., 2017). The core issue affecting the long-term survival of renal transplant recipients lies in the precise regulation of immunosuppressive therapy. The triple immunosuppressive regimen (calcineurin inhibitor [CNI], mycophenolic acid [MPA], and corticosteroids) recommended by The Transplantation Society (TTS) has become the established foundation for maintenance immunosuppression in clinical renal transplantation (KDIGO clinical practice guideline for the care of kidney transplant recipients, 2009). Especially, MPA-class agents play a pivotal role in preventing acute rejection through selective inhibition of T/B lymphocyte proliferation (Allison, 2005; Bhat et al., 2023; Behrend and Braun, 2005). Nevertheless, the clinical application of MPA-class drugs faces substantial challenges: narrow therapeutic window, significant interindividual pharmacokinetic variability, and high incidence of gastrointestinal toxicity (Bergan et al., 2021; Sobiak and Resztak, 2021). Currently, mycophenolate mofetil (MMF) and mycophenolate sodium (MPS), as two principal MPA prodrugs, exhibit distinct clinical profiles due to differences in pharmaceutical formulation despite sharing the same active metabolite (de Winter et al., 2008). The rational selection between these agents has emerged as a critical issue in optimizing immunosuppressive therapy.

Both MMF and MPS are prodrugs of MPA that require *in vivo* conversion to the active metabolite MPA. Their immunosuppressive effects are mediated through inhibition of purine synthesis in immune cells by blocking inosine monophosphate dehydrogenase enzyme activity (Tedesco-Silva et al., 2005). While sharing this common mechanism, their pharmacological divergence originates from distinct delivery systems (Budde et al., 2007a). MMF, an ester derivative produced as cost-effective conventional tablets, undergoes rapid hydrolysis to active MPA in the stomach and proximal small intestine with 94% bioavailability, yet exhibits significant first-pass metabolism and marked plasma concentration fluctuations (Jacqz-Aigrain et al., 2000; Tang et al., 2017). In contrast, MPS exists as a sodium salt of MPA. The enteric-coated MPS (EC-MPS) with delayed-release technology targeting the ileum’s alkaline environment, circumvents gastric acid degradation and minimizes direct mucosal irritation, thereby achieving enhanced gastrointestinal tolerability and more stable pharmacokinetic profiles (Sobiak et al., 2021; Wang et al., 2022). Clinically, MMF excels in rapid onset and cost-effectiveness, whereas EC-MPS demonstrates superior tolerability and pharmacokinetic stability. This pharmacological advantage positions EC-MPS as the preferred option for sensitive populations, including patients with diabetic gastroenteropathy, elderly recipients, and pediatric transplant cohorts, particularly given the frequent gastrointestinal complications observed with MMF in renal transplantation practice (Gabardi et al., 2003).

The area under the concentration-time curve from 0 to 12 h (AUC_0–12h_) is conventionally used to evaluate MPA exposure, with an internationally accepted therapeutic target range of 30–60 mg h·L^-1^ in renal transplant recipients (Chen et al., 2019; Tett et al., 2011). While phase III clinical trials have established therapeutic equivalence between EC-MPS (720 mg twice daily) and MMF (1,000 mg twice daily) in terms of efficacy and safety, significant pharmacokinetic disparities persist. EC-MPS demonstrates higher pre-dose trough concentrations, lower peak concentrations, and prolonged time to maximum concentration compared to MMF, alongside greater absorption-phase variability evidenced (Salvadori et al., 2004; Graff et al., 2016; Budde et al., 2007b). Crucially, EC-MPS exhibits interindividual variability in MPA exposure, rendering standardized dosing regimens suboptimal as evidenced by 20% of patients exceeding the therapeutic window (>60 mg h·L^-1^) and 35% failing to achieve threshold exposure (<30 mg h·L^-1^) under empirical dose adjustment (Budde et al., 2007b; Collins et al., 2020; Kiang and Ensom, 2018). These pharmacodynamic complexities, compounded by the narrow therapeutic index of MPA-based immunosuppressants, mandate the implementation of therapeutic drug monitoring (TDM) as a cornerstone of precision dosing strategies.

The substantial pharmacokinetic variability and definitive concentration-effect correlation of EC-MPS constitute the most compelling rationale for implementing TDM to guide AUC-based dose individualization. However, conventional TDM approaches face practical limitations, particularly the clinical infeasibility of intensive sampling protocols in transplant populations (Hougardy et al., 2016). To address this, limited sampling strategies integrating population pharmacokinetic (popPK) modeling with Bayesian forecasting have been advocated as a pragmatic solution for optimizing EC-MPS dosing regimens (Wang et al., 2022; Fromage et al., 2025). As a superior alternative to classical pharmacokinetic methods, popPK analysis enables precise quantification of inter- and intra-individual variability through sparse sampling, while facilitating identification of clinically significant covariates influencing drug exposure (Duffull et al., 2011). Nevertheless, the external validity of such models may be compromised by center-specific factors including study design heterogeneity, sample size limitations, and analytical platform discrepancies (El Hassani and Marsot, 2023). Rigorous external validation using independent multicenter datasets is therefore mandated prior to clinical implementation across diverse healthcare settings, ensuring robust model generalizability and therapeutic reliability.

Despite the development of numerous popPK models over the past 20 years to characterize the pharmacokinetics of EC-MPS, the external applicability of these models across multicenter settings remains inadequately validated. Systematic evaluation of model transferability not only addresses this knowledge gap but also facilitates identification of center-specific covariates impacting predictive performance. Furthermore, strategic selection of optimal popPK models from existing literature—rather than conducting *de novo* modeling—may represent a resource-efficient approach for personalized dosing guidance. To address these imperatives, this study implemented a comprehensive validation framework using independent datasets to assess the predictive capacity of published popPK models for EC-MPS within triple immunosuppressive regimens in adult renal transplant recipients.

## 2 Methods

### 2.1 Review of published popPK analyses of MPA

A literature search was conducted using PubMed, Embase, and Web of Science databases from their inception to November 2024, following the Preferred Reporting Items for Systematic Reviews and Meta-analyses (PRISMA) guidelines. The search strategy included the following keywords: *“mycophenolic acid” OR “mycophenolate sodium”*, *“population pharmacokinetics” OR “PPK” OR “equation”*, and *“kidney” OR “renal”*. The inclusion criteria were: (1) Studies involving renal transplant recipients; (2) Administration of MPS at therapeutic dosages; (3) popPK studies utilizing a non-linear mixed-effects modeling approach; (4) Publication in English; (5) Full-text availability. The exclusion criteria were (1) Studies not involving MPS; (2) popPK studies incorporating genetic polymorphisms as covariates; (3) Insufficient data for external validation; (4) Duplicate datasets or overlapping cohorts. In cases of dataset overlap, only the most recent study or the one with the largest sample size was retained. Two independent authors screened the titles, abstracts, and full texts of identified articles for eligibility. A third author resolved discrepancies through consensus. It is worth noting that studies incorporating genetic polymorphisms as covariates were excluded because our external validation dataset, derived from routine clinical practice, lacked genetic polymorphism information. This ensured all evaluated models could be fully tested using available covariates.

### 2.2 Study cohort of external evaluation

A total of 31 Chinese renal transplant recipients treated with triple immunosuppressive regimen (CNI + MPA + corticosteroids) at the Affiliated Hospital of Qingdao University from March 2023 to February 2025 were enrolled in this study. For each patient, 6 serial blood samples were collected, resulting in a total of 186 samples. The protocol was approved by the Ethics Committee of the Affiliated Hospital of Qingdao University (Approval Number: QYFY WZLL 30016). All patients received twice-daily 540 mg EC-MPS doses (every 12 h). Following a consistent administration regimen for at least 3 days, 6 serial blood samples were collected per patient: 0.5 h pre-dose, 1, 2, 4, 8, and 12 h post-dose. Heparin sodium served as the catheter anticoagulant during sample collection. The blood samples were then centrifuged to separate the plasma and stored at −20 °C for analysis. MPA concentrations were quantified using a validated liquid chromatograph-mass spectrometer (LC-MS) method with a calibration range of 0.1–20 μg ml^-1^ and a lower quantification limit of 0.1 μg ml^-1^. Additional clinical data encompassing demographic profiles, biochemical parameters, hematological indices, and concomitant medications were retrospectively extracted from medical records.

### 2.3 External predictive ability evaluation

The external validation of the MPA popK model was conducted through goodness-of-fit analysis, prediction error testing based on model predictions, and visual predictive checks based on model simulation. Published popPK models (de Winter et al., 2008; Wang et al., 2022; Sam et al., 2009; Veličković-Radovanović et al., 2015) were reconstructed by incorporating reported structural parameters, with patient medication records and biochemical function data from our hospital serving as input datasets. External validation procedures were executed using NONMEM^®^ (version 7.6.0, ICON Development Solutions, Ellicott City, MD, United States), while R software (version 4.4.1, http://www.r-project.org/) processed the NONMEM output for subsequent analysis. All statistical evaluations and graphical representations were generated using Xpose4 (Version 23.0.0), ensuring comprehensive assessment of model performance across multiple validation approaches.

#### 2.3.1 Goodness of fit analysis

The goodness-of-fit method assesses the proximity and correlation between observed concentrations and predicted concentrations by creating scatter plots of dependent variable-population predicted concentrations (DV-PRED) and dependent variable-individual predicted concentrations (DV-IPRED) (Nanga et al., 2022; Wei et al., 2022). These visualizations facilitate the evaluation of model performance by quantifying the alignment between predicted and actual values, ultimately determining the degree of fit achieved by the predictive model (Tauzin et al., 2019).

#### 2.3.2 Prediction error test

The prediction error test method evaluates model performance by estimating the prediction error (PE) (Equation 1) and the individual prediction error (IPE) (Equation 4), respectively. The median prediction error (MDPE) (Equation 2) and the median individual prediction error (MDIPE) (Equation 5) were used to evaluate the accuracy of prediction. The median absolute prediction error (MAPE) (Equation 3) and the median absolute individual prediction error (MAIPE) (Equation 6) were used to evaluate the precision of prediction (Sheiner and Beal, 1981; Zhang et al., 2023; Mizaki et al., 2023). Moreover, F_20_ and F_30_ represent the percentage of PE within the ±20% and ±30% ranges respectively, while IF_20_ and IF_30_ denote the percentage of IPE within the same respective ranges. These metrics also served as a combined measure of both accuracy and precision (Yang et al., 2022; Huang et al., 2020). For optimal model performance, criteria specify that MDPE or MDIPE should be ≤ 20%, MAPE or MAIPE ≤30%, F_20_ or IF_20_ ≥ 35%, and F_30_ or IF_30_ ≥ 50% (Zhang et al., 2023; Yang et al., 2022; Zhang et al., 2019).

$$\text{PE}=\left(\text{PRED}-\text{OBS}\right)/\text{OBS}\times 100\%$$ (1)

MDPE=median of PE (2)

$$\text{MAPE}=\text{median of }\left|\text{PE}\right|$$ (3)

$$\text{IPE}=\left(\text{IPRED}-\text{OBS}\right)/\text{OBS}\times 100\%$$ (4)

MDIPE=median of IPE (5)

$$\text{MAIPE}=\text{median of }\left|\text{IPE}\right|$$ (6)

where OBS was the observed concentrations, PRED was the population predicted concentrations, IPRED was the individual predicted concentration.

#### 2.3.3 Visual predictive verification

The visual predictive check method employs model parameters to perform 1,000 simulations of the dataset, calculating the 95% confidence intervals for the fifth, 50th, and 95th percentiles of fitted concentrations across different models (Tauzin et al., 2019; Zhang et al., 2023). These intervals are compared with observed concentrations to identify systematic deviations between observed data and the simulated data (Wei et al., 2022). By comprehensively evaluating model fitting effect, deviation degree, accuracy and precision, the MPA popPK model most suitable for our hospital’s patient population was ultimately identified.

## 3 Results

### 3.1 Review of published popPK analyses of MPA

**FIGURE 1:**
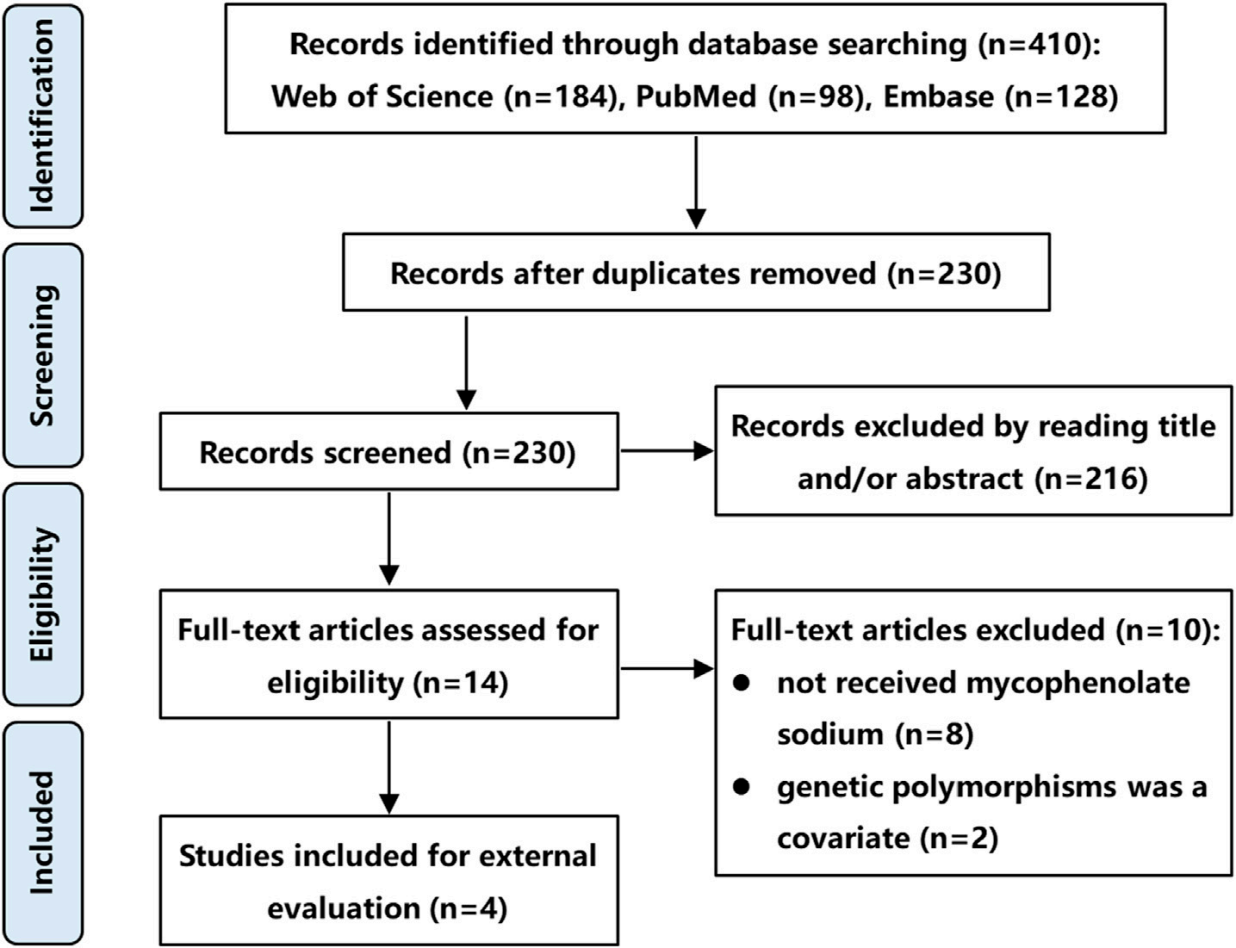
Flow diagram of literature selection process.

Following a systematic literature review, four popPK models investigating the co-administration of MPS and tacrolimus were identified and selected for external validation, with the search strategy detailed in Figure 1. These models (designated Model 1 (de Winter et al., 2008), Model 2 (Sam et al., 2009), Model 3 (Veličković-Radovanović et al., 2015), and Model 4 (Wang et al., 2022) were exclusively developed in kidney transplant recipients. It is worth noting that in de Winter et al. (2008), the construction and optimization process of the popPK model for MPS and MMF were addressed. Since MPS was used in our external validation patient cohort, and MMF was not involved in our study, the ultimately optimized MPS model was incorporated into our study (designated Model 1) for external evaluation. Model 1 was a multinational multicenter study, while Models 2-4 were single-center studies. Regarding structural characteristics, Model 4 employed a one-compartment models whereas Models 1-3 utilized two-compartment models. Basic information and parameter information for each model were systematically presented in Tables 1, 2.

**TABLE 1:** Basic information of MPA popPK models.

Model	Race or ethnicity	Patients (M/F)	Age (years)	Weight (kg)	Dosing of EC-MPS	Combined drugs	Samples	Detection methods	Software	Verification	Refs.
1	Caucasian	167 (117/50)	45 (21–79)	76 (40–124)	674 (337–1,348) mg·day^-1^	Tacrolimus, Cyclosporin A, Everolimus	2,309	HPLC	NONMEM	GOF BS VPC	de Winter et al. (2008)
2	American	18 (18/0)	46 (18–63)	85.7 (57–133)	720 (720–1,440) mg·day^-1^	Tacrolimus, Cyclosporin A, Prednisolone	232	HPLC	NONMEM	GOF VPC	Sam et al. (2009)
3	Serbian	70 (48/22)	42.97 (21–70)	75.33 (53–113)	1,028.57 (720–1,440) mg·day^-1^	Tacrolimus, Cyclosporin A, Prednisolone, Omeprazole, Bisoprolol, Carvedilol, Nitrendipine	90	HPLC	NONMEM	GOF VPC	Veličković-Radovanović et al. (2015)
4	Chinese	96 (52/44)	13.3 (4.3–18)	39 (15–67)	10.5 (3.4–24.0) mg·kg^-1^·day^-1^	Tacrolimus, Cyclosporin A	384	EMIT	Monolix	GOF VPC	Wang et al. (2022)

Note: Data were presented as median (range). M (Male), F (Female), HPLC (high-performance liquid chromatography), EMIT (enzyme-multiplied immunoassay technique), BS (bootstrap), GOF (goodness of fit), VPC (visual predictive check).

**TABLE 2:** Parameter information of MPA popPK models.

Model	Compartment	Structural model formula	Individual variation	Residual variation	Refs.
1	Two	CL (L·h^-1^) = 16 V_2_ (L) = 40 V_3_ (L) = 518 Q (L·h^-1^) = 22 KA (h^-1^) = 3 ALAG1_MD1_ (h) = 0.95 ALAG1_MD2_ (h) = 1.88 ALAG1_MD3_ (h) = 4.83 ALAG1_ED_ (h) = 9.04 POP with ALAG1_MD1_ = 0.51 POP with ALAG1_MD2_ = 0.32 POP with ALAG1_MD3_ = 0.17	CL: 0.39 V_2_: 1 V_3_: 4.9 Q: 0.78 KA: 1.87 ALAG1_MD_: 0.08 ALAG1_ED_: 0.4	Add = 0.39	de Winter et al. (2008)
2	Two	CL (L·h^-1^) = 10.6 V_2_ (L) = 25.9 V_3_ (L) = 39.6 Q (L·h^-1^) = 8.11 KA (h^-1^) = 0.673	CL: 0.214 V_2_: 0.878 V_3_: 2.39	ProP = 0.699	Sam et al. (2009)
3	Two	CL (L·h^-1^) = 0.741 + 0.0804 × AGE + 0.00165 × DD + 1.12 × NIF V (L) = 0.653 VSS (L) = 801 Q (L·h^-1^) = 52.1 KA (h^-1^) = 4.07 ALAG1 (h) = 0.21	CL: 0.25 V: 0.24 VSS: 12.41 Q: 2.16 KA: 1.44 ALAG1: 0.35	ProP = 0.35	Veličković-Radovanović et al. (2015)
4	One	CL (L·h^-1^) = 4.28 × (BSA/1.23)^1.3^ V (L) = 3.73 KA (h^-1^) = 0.123 D_2_ (h) = 2.9 F1 = 0.553 ALAG1 (h) = 8.45 diff_ALAG2_ = 5.78	CL: 0.481 V: 0.337 KA: 0.885 D_2_: 2.31 F1: 0.513 ALAG1: 0.576 diff_ALAG2_ : 0.941	a = 0.0631 b = 0.199	Wang et al. (2022)

Note: CL (clearance), V (apparent volume of distribution), V_2_ (volume of distribution of the central compartment), V_3_ (volume of distribution of the peripheral compartment), VSS (volume of distribution at steady-state), Q (intercompartmental clearance), KA (absorption rate constant), MD (morning dose), ED (evening dose), ALAG1_MD1_ (lag-time for people of group MD1), ALAG1_MD2_ (lag-time for people of group MD2), ALAG1_MD3_ (lag-time for people of group MD3), ALAG1_ED_ (lag-time for people of group ED), POP (part of the population), DD (total daily dose of mycophenolate sodium, mg·day^-1^), NIF (co-medication with nifedipine), BSA (body surface area), D2 (Duration for zero-order absorption), F1 (fraction for first-order absorption), ALAG1 (lag-time for first-order absorption), ALAG2 (lag-time for zero-order absorption), diff_ALAG2_ (the time by which ALAG2 is longer than ALAG1), Add (additive error), ProP (proportional error), a and b (residual error model parameters from the equation C_obs_ = C_pred_ + sqrt [a^2^ + (b·C_pred_)^2^]·ε).

### 3.2 External evaluation cohort

Blood drug concentration monitoring data of 31 renal transplant patients were included in this study, including 22 male patients and 9 female patients, with an average age of 39.29 ± 10.77 years old. All 31 patients were treated with tacrolimus and hormone therapy. More basic demographic information and clinical indicators are presented in Table 3.

**TABLE 3:** Basic demographic information and clinical indicators of including patients.

Characteristics	Number or mean ± SD	Median (range)
No. of patients (Male/Female)	31 (22/9)	—
Age (years)	39.29 ± 10.77	37 (19–60)
Height (cm)	171.03 ± 9.14	170 (153–189)
Body weight (kg)	171.03 ± 9.14 kg	69 (40.15–100.33)
Serum albumin (g·L^-1^)	36.27 ± 3.87	35.6 (29.3–42.6)
Serum creatinine (umol·L^-1^)	232.29 ± 238.03	136.74 (72.9–1,136.08)
ALT (U·L^-1^)	18.10 ± 9.38	17 (4–42)
AST (U·L^-1^)	18.55 ± 8.49	17 (7–56)
Urea (mmol·L^-1^)	18.90 ± 12.50	13.6 (8.1–64.6)
Hemoglobin (g·L^-1^)	95.45 ± 17.94	94 (64–132)
BSA (m^2^)	1.80 ± 0.23	1.81 (1.35–2.30)
EC-MPS dose (g·day^-1^)	1.08	1.08
Tacrolimus dose (mg/day)	7.15 ± 2.10	7.75 (0.4–11)
Corticosteroids dose (mg/day)	90.83 ± 168.58	20 (12–500)

Note: ALT (alanine transaminase), AST (aspartate transaminase), BSA (body surface area), EC-MPS (enteric-coated mycophenolate sodium).

### 3.3 External predictability evaluation

#### 3.3.1 Goodness of fit diagnosis

**FIGURE 2:**
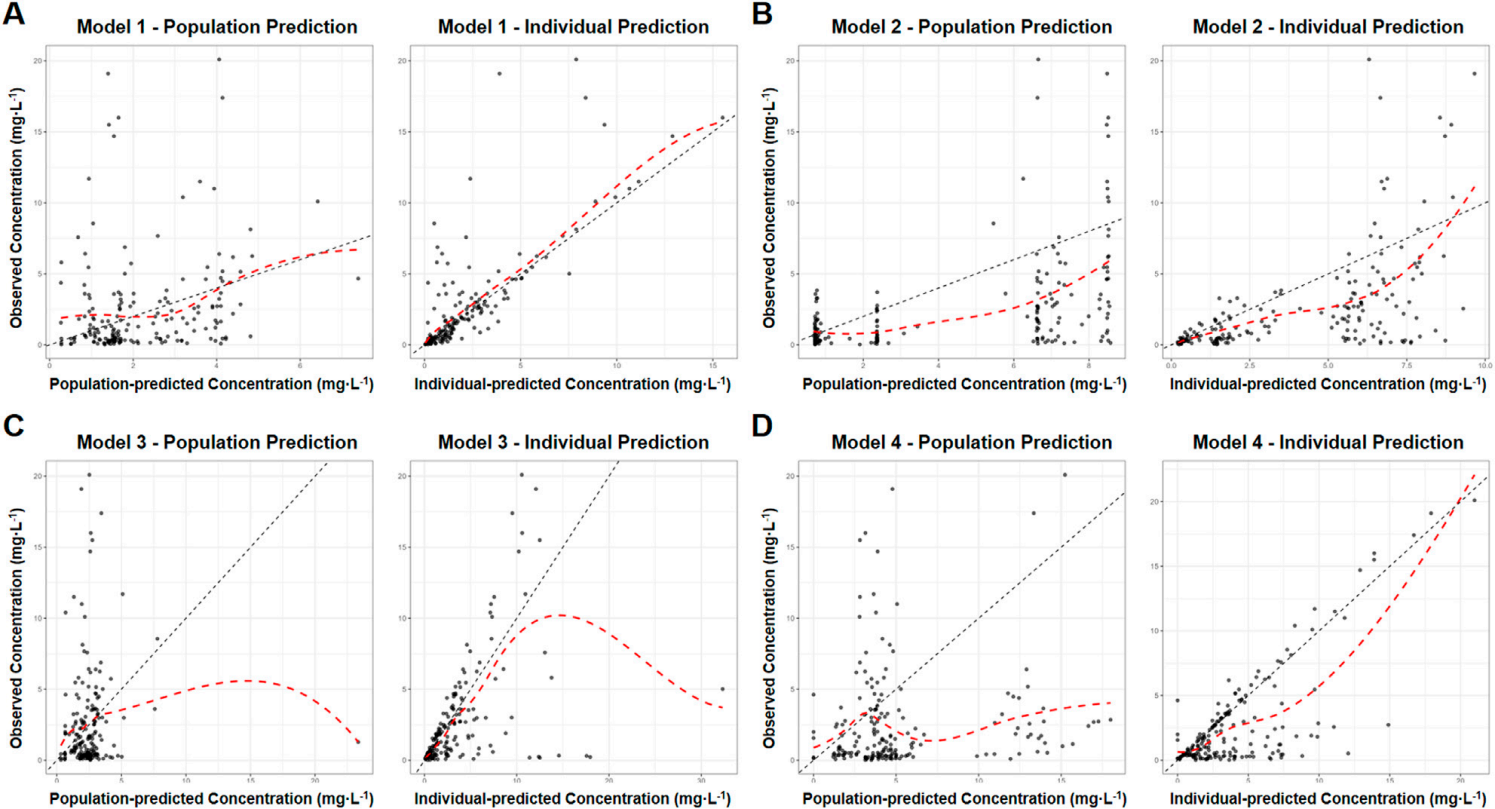
Goodness of fit graphs of MPA popPK models for renal transplant patients in our hospital. **(A)** Model 1. **(B)** Model 2. **(C)** Model 3. **(D)** Model 4. (Left: population prediction, DV-PRED scatter plot. Right: individual predictions, DV-IPRED scatter plot. Black dashed lines: the reference line (Y = X). Red dashed lines: trend lines).

The MPA concentration was predicted using published popPK models based on renal transplant patients’ medication data. The scatter plots of dependent variable vs. population-predicted (DV-PRED) and dependent variable vs. individual-predicted (DV-IPRED) for each model were presented in Figure 2. The scatter plot on the left depicted population prediction. Each point corresponded to an observed data point plotted against its population prediction. The scatter plot on the right depicted individual predictions. Similarly, each point represented an observed data point plotted against its individual prediction. Black dashed lines indicating the reference line (Y = X) and red dashed lines representing trend lines. Trend lines were calculated using locally estimated scatterplot smoothing (LOESS) regression and generated by the R software. Better model fit was demonstrated when predicted values closely approximate actual observations, as evidenced by higher concordance between trend lines and the reference line.

The left DV-PRED scatter plot showed that the coincidence degree between the trend line and the reference line for the population data in Models 1-4 were relatively low. There were almost no overlapping sections between the trend line and the reference line, and the scatter distribution was not uniform enough. All R^2^ values were lower than 0.3, and the regression coefficients and residuals of the linear fitting were not ideal either, suggesting that the prediction performance of the model was poor.

The right DV-IPRED scatter plot indicated that the individual predicted values of Model 1 had a relatively good correlation with the actual observed values. The coincidence degree between the trend line and the reference line was relatively high and the linear regression coefficient was 1.15, and R^2^ value was 0.64. Scattered points were symmetrically distributed and clustered around the diagonal, reflecting reasonable aggregation trends and dispersion patterns. Nevertheless, the prediction performance of the remaining models was relatively poor. Models 2 and 4 showed partial alignment of trend line and the reference line at extreme concentrations but exhibit offsets and scattered mid-concentration data points, The prediction effects were not good with regression coefficients of 0.71/0.74, and R^2^ values of 0.31/0.55, respectively. Model 3 aligned only at low concentrations, deviated at higher concentrations with scattered data points, and featured a distinct outlier causing trend line displacement. The regression coefficient was 0.44, and R^2^ value was 0.26, all indicating inadequate predictive accuracy.

#### 3.3.2 Prediction error test

**FIGURE 3:**
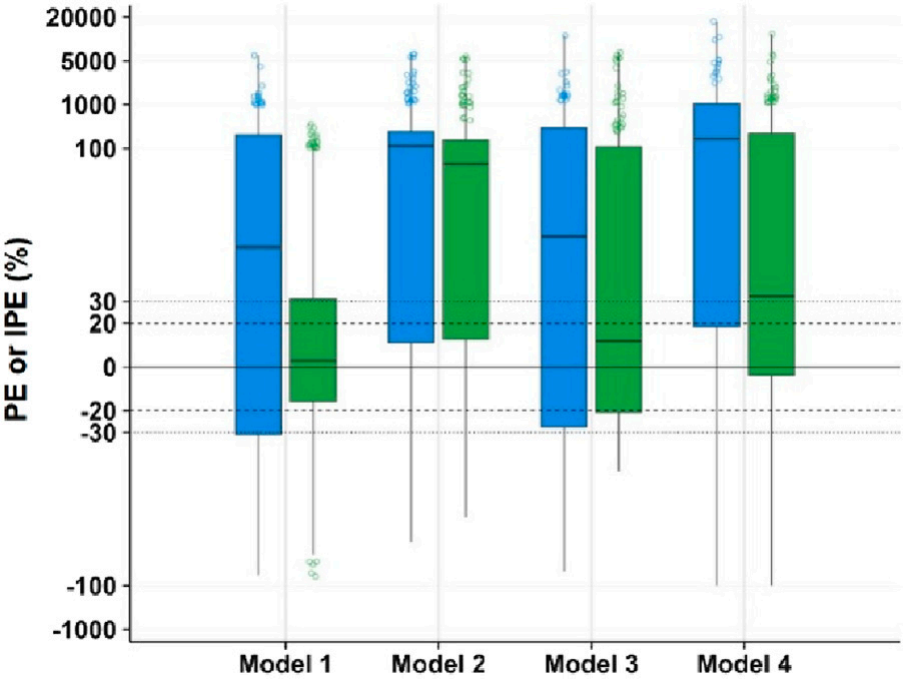
Box plot of prediction error of MPA popPK models for renal transplant patients in our hospital. (Blue box: population predicted. Green box: individual predicted. The solid line in box: median. Dashed lines: ±20% prediction error thresholds. Dotted lines: ±30% prediction error thresholds).

The prediction error analysis results were presented in Figure 3 and Table 4. In the graphical representation, the solid black line denoted the zero-error reference, while dashed and dotted lines demarcated the ±20% and ±30% prediction error thresholds, respectively. In boxplots, the blue boxes represented the population prediction error and the green boxes represented the individual prediction error. Closer alignment of the median line (box solid line) with the zero-error reference indicated higher prediction accuracy, whereas narrower box widths reflected better precision of the model predictions.

**TABLE 4:** Prediction error test of MPA popPK models for renal transplant patients in our hospital.

Model	MDPE (%)	MAPE (%)	F_20_ (%)	F_30_ (%)	MDIPE (%)	MAIPE (%)	IF_20_ (%)	IF_30_ (%)
1	54.76	81.51	11.29	17.20	3.05	25.00	44.09	56.45
2	150.62	150.62	10.22	13.44	92.78	92.78	15.59	23.66
3	59.72	82.32	11.29	18.28	11.80	33.33	25.27	44.09
4	300.21	300.21	5.91	10.22	32.32	50.47	39.25	45.70

The population prediction error test showed that the distance between the prediction error box of each model group and the zero line was relatively far. MDPE, MAPE, F_20_ and F_30_ also did not meet the above standards, indicating that the fitting effect of these several models at the population error prediction level was poor.

In the individual prediction error box diagram, the solid line of the box in Model 1 was closest to the zero line, with the highest accuracy. Meanwhile, its box was the narrowest and had the best precision. The MDIPE of Model 1 ≤ 20%, the MAIPE ≤ 30%, the IF_20_ > 35%, and the IF_30_ > 50%, indicating that Model 1 had a good fitting effect at the individual error prediction level. Furthermore, the solid lines of the boxes in Model 3 and Model 4 were relatively close to the zero line. The MDIPE of Model 3 was within 20%, and the IF_20_ of Model 4 was greater than 35%, while the remaining standards failed to meet the requirements. Moreover, the distance between the group prediction error box and the zero line in Model 2 was relatively far, MDIPE, MAIPE, IF_20_ and IF_30_ did not meet the above standards either, indicating that the fitting effect of Model 2 at the individual error prediction level was poor.

#### 3.3.3 Visual predictive verification

**FIGURE 4:**
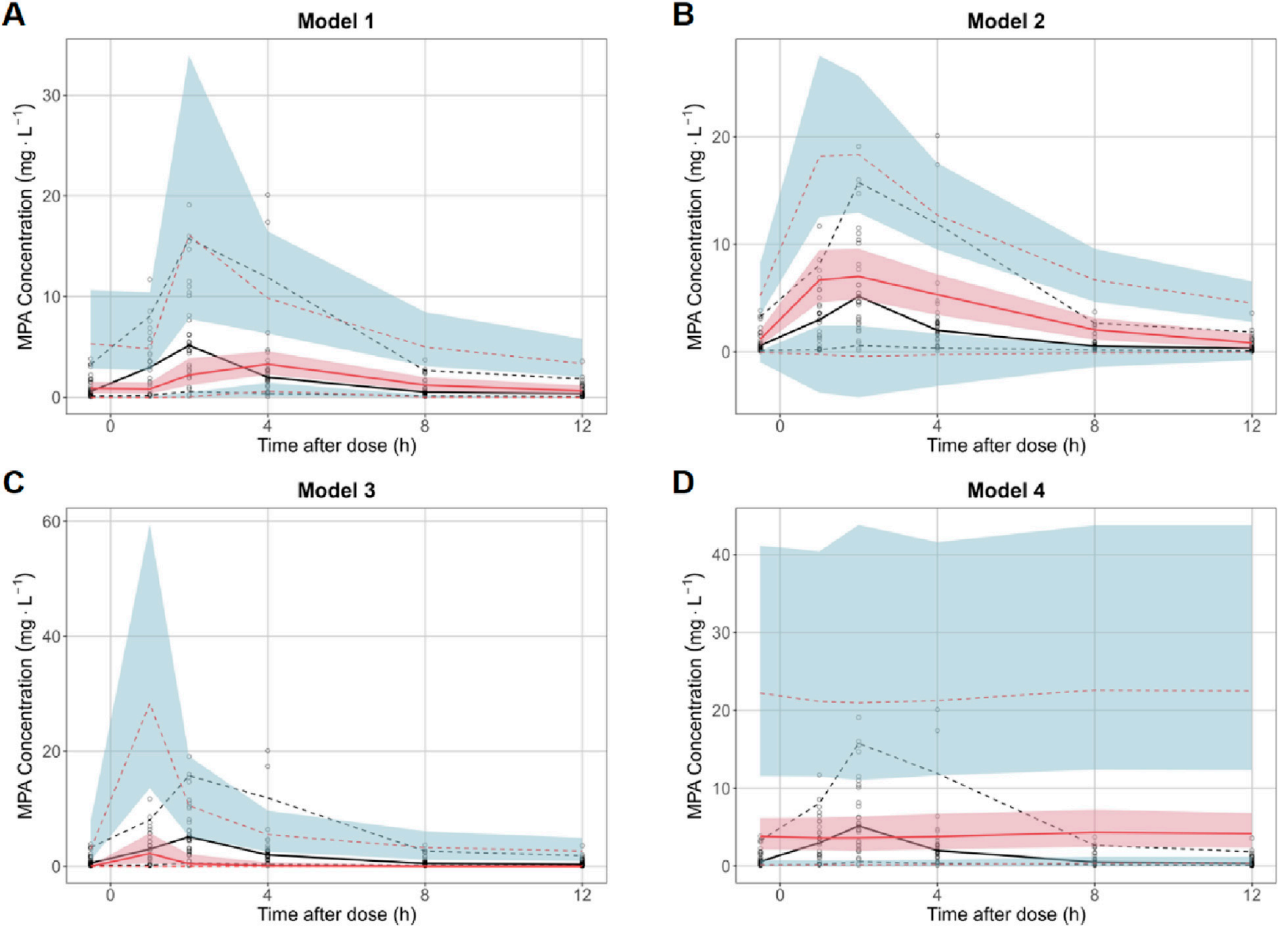
Visual predictive check of MPA popPK models for renal transplant patients in our hospital. **(A)** Model 1. **(B)** Model 2. **(C)** Model 3. **(D)** Model 4. (Black hollow dot: observed concentrations. Black curves at top, middle and bottom: 95th, 50th and fifth percentiles of the observed concentrations. Red curves at top, middle and bottom: 95th, 50th and fifth percentiles of the simulated concentrations. Shaded areas at top (blue), middle (red) and bottom (blue): the 95% confidence intervals for the 95th, 50th and fifth percentiles of the simulated concentration).

The model and dataset were subjected to 1,000 simulations using the Visual Predictive Check (VPC) module in NONMEM software, with results shown in Figure 4. The plot displays time after dose (h) on the x-axis versus MPA concentration (mg·L^-1^) on the y-axis. Data points represent actual observed concentrations, while three black lines (from top to bottom) correspond to the 95th, 50th, and fifth percentiles of observed data. Three red lines (from top to bottom) correspond to the 95th, 50th and fifth percentiles of the simulated concentrations, while the shaded areas represent their 95% confidence intervals. Enhanced model performance is demonstrated by closer alignment between observed percentiles (black lines) and their corresponding simulated confidence intervals (shaded regions), reflecting improved goodness-of-fit.

VPC revealed significant inter-model performance variation, with Model 1 demonstrating superior concordance between observed and simulated concentrations across the fifth, 50th, and 95th percentiles, encompassing 98.4% of observations within the 95% simulation confidence bands. In contrast, Models 2-4 exhibited suboptimal distributional alignment, characterized by reduced inclusion rates of observed percentiles within their respective prediction intervals. Notably, Model 4 manifested substantial predictive bias, displaying systematically inflated simulated concentrations with excessively wide confidence intervals that diverged markedly from the empirical data distribution. This pronounced discordance suggests fundamental limitations in Model 4’s structural specification or parameter estimation.

## 4 Discussion

As the two principal prodrugs of MPA, MMF and MPS demonstrate distinct clinical profiles despite sharing identical active metabolites, the difference attributable to their distinct prodrug structures and pharmaceutical formulations (Tedesco-Silva et al., 2005; Sobiak et al., 2021). To date, popPK models for MMF in renal transplant recipients have been extensively developed and externally validated (Zhang et al., 2019). In contrast, limited popPK research exists for MPS, with its external validity remaining poorly characterized. To our knowledge, this study represents the first comprehensive external validation of published MPS popPK models using an independent clinical dataset. While the validation dataset originated from a single center and had limited sample size, this investigation provides critical insights to advance research on MPS popPK characteristics.

This study evaluated the predictive performance of four published MPS popPK models using TDM data from our institutional cohort. Results demonstrated deficient predictive accuracy of existing models, with all failing to meet validation standards. Both population prediction analyses through goodness-of-fit diagnostics and prediction error testing revealed poor agreement between predicted and observed values. Individual predictions showed marginally better performance for Model 1, achieving composite precision indices (IF_20_ > 35% and IF_30_ > 50%), though the DV-IPRED scatter plots exhibited systematic bias and reduced overlap in high-concentration ranges due to data dispersion. In addition, the results of the VPC test based on model simulation show that the fitting results of Model 1 is relatively good, the distribution of the observed data and the simulation data is relatively close. Besides, for majority models, the observed concentration value data that fall within the confidence interval of the simulated data are relatively few, the deviation is large, and the prediction effect is poor.

The observed discrepancies between published model predictions and measured concentrations in our cohort may stem from clinical variables and methodological considerations, including interindividual pathophysiological heterogeneity, analytical variability in MPA quantification methodologies and the differential selection of pharmacokinetic modeling approaches.

Demographic characteristics, particularly race and age, emerge as significant covariates influencing MPA pharmacokinetic PK parameters, with sample size additionally affecting predictive accuracy. Variability in these key determinants across published models, including racial composition, age distribution, and cohort size, may introduce bias in popPK parameter estimation, ultimately compromising model predictive performance (Catić-Đorđević et al., 2021; Tornatore et al., 2022; Lestini et al., 2015). Comparative analysis reveals: Model 1 comprise 2,309 TDM samples from multi-regional Caucasian adults (mean age: 45 years), Model 2 comprise 232 TDM samples from American adults (mean age: 46 years), Model 3 comprise 90 TDM samples from Serbian adults (mean age: 42.97 years) and Model 4 comprise 384 TDM samples from Chinese pediatric patients (mean age: 13.3 years). Our external validation cohort comprised 186 Chinese adult TDM samples (mean age: 39.29 years). The enhanced predictive accuracy of Model 1 likely reflects its larger sample size providing greater statistical power. Notably, the complexity of race-specific metabolic variations may be the reason for the poor prediction of Model 2 and Model 3. And Model 4’s suboptimal performance may stem from fundamental PK differences between pediatric and adult populations.

Notably, the published models incorporated different analytical methods for detecting MPA blood concentration, including HPLC, HPLC/UV, and EMIT, while the external cohort dataset of our hospital adopts HPLC-MS. Variations in the accuracy and precision across these detection methodologies may introduce variability that could compromise the model’s predictive performance and stability in concentration estimation.

The selection of structural models, computational platforms, and significant covariates differs among published popPK analyses, potentially introducing variability in parameter estimation and influencing the predictive accuracy of the final population pharmacokinetic model. Research demonstrates that structural model specification directly affects derived pharmacokinetic parameters (Yu et al., 2017). Among the evaluated models, Models 1–3 implemented a two-compartment disposition model, whereas Model 4 utilized a single-compartment approximation. Given MPA’s biphasic elimination characteristics and tissue distribution profile, the two-compartment configuration appears physiologically more plausible, potentially explaining Model 4’s substantial prediction error and inflated variability estimates (de Winter et al., 2009). The enteric-coated formulation of MPS employs pH-dependent release kinetics targeting ileal absorption, thereby avoiding gastric degradation and minimizing mucosal irritation (Wang et al., 2022). This pharmacokinetic profile necessitates explicit incorporation of absorption lag-time (ALAG) in modeling (de Winter et al., 2008). The published models exhibit significant ALAG variations: Model 1 (∼2 h), Model 2 (not incorporated), Model 3 (0.21 h), and Model 4 (8.45 h). Model 1’s superior goodness-of-fit likely stems from its appropriate ALAG parameterization, which aligns with the drug’s known gastrointestinal transit dynamics.

Overall, this investigation utilized TDM data for MPA following MPS administration, collected through routine clinical monitoring protocols, thereby providing an objective representation of real-world pharmacological variability. The dataset encompassed trough, intermediate, and peak concentrations measured at standardized sampling intervals, enabling systematic evaluation of published models’ capacity to characterize pharmacokinetic profiles across critical temporal phases. The published models exhibit significant variability and unsatisfactory predictive performance, indicating that TDM remains an essential requirement for the clinical application of MPS. To advance individualized medication for MPS based on popPK, future research must prioritize the investigation of potential covariates. This will enable identification of key factors influencing MPS model predictability and facilitate the development of a popPK model suitable for patients in our hospital.

## Data Availability

The original contributions presented in the study are included in the article/Supplementary Material, further inquiries can be directed to the corresponding authors.
